# Association of four genetic variants with colorectal cancer in Kazakhstan population

**DOI:** 10.18632/oncotarget.28070

**Published:** 2021-10-12

**Authors:** Yevgeniya Kolesnikova, Dmitriy Babenko, Irina Kadyrova, Svetlana Kolesnichenko, Lyudmila Akhmaltdinova, Ilya Korshukov, Naylya Kabildina, Valentina Sirota, Vera Zhumaliyeva, Dana Taizhanova, Dmitriy Vazenmiller, Anar Turmukhambetova

**Affiliations:** ^1^Department of Biomedicine, Karaganda Medical University, Karaganda 100008, Kazakhstan; ^2^Research and Scientific Center, Karaganda Medical University, Karaganda 100008, Kazakhstan; ^3^Shared Resource Laboratory, Karaganda Medical University, Karaganda 100008, Kazakhstan; ^4^Department of Oncology and Radiation Diagnostics, Karaganda Medical University, Karaganda 100008, Kazakhstan; ^5^Department of Internal Medicine, Karaganda Medical University, Karaganda 100008, Kazakhstan; ^6^Department of Obstetrics, Gynecology and Perinatology, Karaganda Medical University, Karaganda 100008, Kazakhstan; ^7^Department of Strategic Development and Science, Karaganda Medical University, Karaganda 100008, Kazakhstan

**Keywords:** colorectal adenocarcinoma, rs10795668, rs1035209, rs11190164, rs10506868

## Abstract

The study was conducted to search for polymorphisms located in the 10th chromosome associated with colorectal adenocarcinoma in representatives of the Kazakhstan population. Study was performed with 282 colorectal cancer (CRC) patients and 159 controls. Genotyping of SNPs was performed by QuantStudio 12K Flex PCR. For four significant SNPs inheritance model analysis was performed. Increasing risk of CRC was noted for rs10795668 in log-additive model (OR = 1.45, 95% CI: 1.05–1.99, *p* = 0.023); for rs1035209 in log-additive model (OR = 1.79, 95% CI: 1.18–2.72, *p* = 0.003); for rs11190164 in log-additive model (OR = 1.67, 95% CI: 1.17–2.38, *p* = 0.004). Decreasing risk of CRC was noted for rs10506868 in log-additive model (OR = 0.56, 95% CI: 0.37–0.85, *p* = 0.006). We detected SNPs that are associated with CRC risk in the Kazakhstan population.

## INTRODUCTION

On a worldwide scale, colorectal cancer (CRC) affects more than 1.9 million patients annually, and more than 935,000 die from the disease [1], which brings CRC third rank by incidence and second rank by mortality among other cancers. There are great differences among the countries and regions in terms of incidence and mortality rates. That could be considered as a measure of socioeconomic development, as far as the rates have the tendency to come after rising human development index (HDI), presumably leading to modify lifestyle factors and diet. Another side is the affordable healthcare policies, making serious distinctions between countries.

In Kazakhstan, a country-wide colorectal screening program was started in 2011, and followed by a smooth rising in incidence and decrement of mortality. The proportion between I–II, III, IV stages among incident cases also was changed over the years, with predominant disease detection at early stages [2].

According to Globocan (2020), Kazakhstan has the highest estimated age-standardized incidence (13.5 per 100 000) and mortality (7.5 per 100 000) rates in the South-Central Asia region [3]. Such discrepancy could be explained by the lack of population-based screening programs in the region. Most countries of the region present no such programs, some settling just pilot projects.

Despite strong hereditary components, most cases of CRC are sporadic and develop slowly over several years. Nowadays studied a genetic variation of single nucleotide polymorphisms (SNPs) and environmental risk factors are considered the etiology of CRC [4]. However, SNPs can vary in ethnic groups and personally adding a share of probability to the development of pathology.

The study was conducted to search for polymorphisms associated with the development of colorectal adenocarcinoma in representatives of the Kazakhstan population. A significant number of polymorphisms associated with colorectal cancer were located in the 10th chromosome. The article aimed to determine the association between SNPs in chromosome 10 and CRC risk in the Kazakhstan population.

## RESULTS

### Study participants

The details of selected characteristics of all subjects were presented in Table 1. We enrolled 441 participants, including 282 patients (149 males, 133 females) and 159 controls (83 males, 76 females) in this study, the mean age of the subjects was 64.97 ± 10.77 years for cases and 46.59 ± 13.58 years for controls. All of the cancers included adenocarcinoma.

**Table 1:** General characteristics of CRC subjects and healthy controls

Group	Case		Control	
Gender	Count	Percentage	Count	Percentage
Male	149	53%	83	52%
Female	133	47%	76	48%
Mean age ± SD	64.97 ± 10.77		46.59 ± 13.58	

### Hardy–weinberg equilibrium and alleles of selected SNPs

A total of 12 SNPs were successfully genotyped in all participants, but not all of the tested SNPs were in accordance with Hardy–Weinberg equilibrium (HWE) in the control group. Thus, 4 SNPs: rs11255841 (HWE *p* = 0.017), rs4948317 (HWE *p* < 0.001), rs704017 (HWE *p* = 0.031), and rs11196172 (HWE *p* = 0.045) did not reach the critical value *p* ≥ 0.05 for HWE in control and were excluded from further analysis. Table 2 presents a list of the studied polymorphisms, their location, and position on the chromosome, possible functions in genes and the achieved level of significance of the exact test for HWE in the control group.

**Table 2:** General characteristics of CRC subjects and healthy controls

SNP ID	Band	Position	Gene	Predicted function	HWE
rs10795668	10p14	8659256			0.362
rs11255841	10p14	8697617			0.017
rs4948317	10q21.1	58811675	BICC1	Intronic	<0.001
rs704017	10q22.3	79059375	ZMIZ1-AS1	non-coding intronic	0.031
rs12412391	10q24.2	99529178	LINC01475	non-coding intronic/	0.445
rs1035209	10q24.2	99585609			0.129
rs11190164	10q24.2	99591947			1.000
rs4919687	10q24.32	102835491	CYP17A1	non-coding/intronic	0.509
rs12241008	10q25.2	112520943	VTI1A	intronic/non-coding intronic	1.000
rs10506868	10q25.2	112559621	VTI1A	Intronic	0.213
rs11196172	10q25.2	112967084	TCF7L2	Intronic	0.045
rs1665650	10q25.3	116727589	HSPA12A	Intronic	0.131

Minor allele frequency, effect alleles, an association between SNPs and risk of CRC in the allelic model analysis were showed in Table 3.

**Table 3:** Minor allele frequency, effect alleles, an association between SNPs and risk of CRC in allelic model analysis

SNP ID	Base change	Minor Allele	MAF Control	MAF Case	Effect allele	OR^a^	*p*-value (*χ*^2^)	FDR
rs10795668	G/A	A	0.39	0.3	G	1.48 95% CI: 1.07 to 2.05	0.019	0.038^*^
rs12412391	A/G	G	0.35	0.38	G	1.16 95% CI: 0.84 to 1.59	0.378	0.432
rs1035209	C/T	T	0.12	0.2	T	1.79 95% CI: 1.18 to 2.71	0.006	0.016^*^
rs11190164	A/G	G	0.18	0.27	G	1.68 95% CI: 1.18 to 2.39	0.005	0.02^*^
rs4919687	G/A	A	0.24	0.29	G	0.8 95% CI: 0.58 to 1.11	0.185	0.296
rs12241008	T/C	C	0.18	0.15	C	0.78 95% CI: 0.53 to 1.15	0.212	0.283
rs10506868	C/T	T	0.16	0.09	T	0.53 95% CI: 0.34 to 0.81	0.004	0.032^*^
rs1665650	C/T	T	0.26	0.27	T	1.04 95% CI: 0.75 to 1.45	0.8	0.8

A Noneffect alleles were considered as references. ^*^statistically significant *p*-value (*p* ≤ 0.05).

Comparison of minor allele frequency (MAF) distribution in Kazakhstan population and among other populations showed similar distribution to East Asian population for rs10795668 (Kazakhstan population control group −0.39, EAS −0.37) and for rs4919687 (Kazakhstan population control group −0.24, EAS −0.25). For rs1035209 (Kazakhstan population control group −0.12) and rs10506868 (Kazakhstan population control group −0.16) MAF was close to South Asian population (SAS –0.09) and (SAS −0.21) respectively. In addition, in two cases MAF for rs12412391 (Kazakhstan population control group −0.35) and rs11190164 (Kazakhstan population control group −0.18) was close to American population (AMR −0.33) and (AMR −0.17) respectively. For rs12241008 MAF was 0.18 in Kazakhstan population control group and close to African population AFR −0.21.

We suggested that the minor allele of each SNP was not always a risk factor for CRC. For rs12412391, rs1035209, rs11190164, rs12241008, rs10506868 and rs1665650 minor allele coincide with effect allele. Therefore, for allelic model analysis and inheritance model analysis non-effect alleles were considered as references [5–10].

Following SNPs were found to be associated with CRC and its risk using the FDR adjustment for *p* values of *χ*^2^ test was calculated: rs10795668, OR = 1.48, 95% CI: 1.07 to 2.05, *p* = 0.038; rs1035209, OR = 1.79, 95% CI: 1.18 to 2.71, *p* = 0.016; rs11190164, OR = 1.68, 95% CI: 1.18 to 2.39, *p* = 0.02. Allelic model analysis of rs10506868 showed reducing the risk of CRC development: OR = 0.53, 95% CI: 0.34 to 0.81, *p* = 0.032.

### Inheritance models

For four significant polymorphisms according to allelic model analysis, inheritance model analysis was performed.

The data obtained allow us to speak about decreasing risk of CRC in the log-additive model for rs10506868, OR = 0.56, 95% CI: 0.37–0.85, *p* = 0.006.

Increasing risk of CRC was noted in rs10795668 log-additive model OR = 1.45, 95% CI: 1.05–1.99, *p* = 0.023. For rs1035209 in dominant model, genotypes CT and TT OR = 1.67, 95% CI: 1.05–2.64, *p* = 0.027, in log-additive model OR = 1.79, 95% CI: 1.18−2.72, *p* = 0.003. For rs11190164 in dominant model, genotype AG and GG, OR = 1.78, 95% CI: 1.17–2.71, *p* = 0.007), in log-additive model OR = 1.67, 95% CI: 1.17–2.38, *p* = 0.004. Genetic analysis results with inheritance models were shown in Table 4.

**Table 4:** Relationship between SNPs and colorectal cancer risk under multiple models of inheritance

Rs	Genotype	Control	Case	OR 95 CI	*p*-value	OR 95 CI adj. by age and gender	*p*-value adj. by age and gender
rs10795668	Dominant						
	A/A	22 (16.9%)	21 (10.1%)	1	0.07	1	0.851
	A/G-G/G	108 (83.1%)	187 (89.9%)	1.81 [0.95–3.45]		1.09 [0.45–2.66]	
	Recessive						
	A/A-A/G	79 (60.8%)	104 (50%)	1	0.053	1	0.032
	G/G	51 (39.2%)	104 (50%)	1.55 [0.99–2.42]		1.93 [1.05–3.53]	
	log-Additive						
	0,1,2	130 (38.5%)	208 (61.5%)	1.45 [1.05–1.99]	0.023	1.43 [0.93–2.2]	0.101
rs1035209	Dominant						
	C/C	109 (75.2%)	156 (64.5%)	1	0.027	1	0.436
	C/T-T/T	36 (24.8%)	86 (35.5%)	1.67 [1.05–2.64]		1.28 [0.68–2.4]	
	Recessive						
	C/C-C/T	145 (100%)	230 (95%)	1	0.005	1	0.007
	T/T	0 (0%)	12 (5%)	NA [0-NA]		NA [0-NA]	
	log-Additive						
	0,1,2	145 (37.5%)	242 (62.5%)	1.79 [1.18–2.72]	0.003	1.48 [0.85–2.59]	0.16
rs11190164	Dominant						
	A/A	98 (67.1%)	140 (53.4%)	1	0.007	1	0.154
	A/G-G/G	48 (32.9%)	122 (46.6%)	1.78 [1.17–2.71]		1.52 [0.85–2.72]	
	Recessive						
	A/A-A/G	141 (96.6%)	242 (92.4%)	1	0.076	1	0.872
	G/G	5 (3.4%)	20 (7.6%)	2.33 [0.86–6.35]		1.1 [0.34–3.56]	
	log-Additive						
	0,1,2	146 (35.8%)	262 (64.2%)	1.67 [1.17–2.38]	0.004	1.34 [0.83-2.16]	0.222
rs10506868	Dominant						
	C/C	105 (71.9%)	213 (83.2%)	1	0.008	1	0.061
	C/T-T/T	41 (28.1%)	43 (16.8%)	0.52 [0.32–0.84]		0.53 [0.27–1.03]	
	Recessive						
	C/C-C/T	140 (95.9%)	252 (98.4%)	1	0.124	1	0.651
	T/T	6 (4.1%)	4 (1.6%)	0.37 [0.1–1.33]		0.66 [0.11–3.93]	
	log-Additive						
	0,1,2	146 (36.3%)	256 (63.7%)	0.56 [0.37–0.85]	0.006	0.6 [0.34–1.06]	0.08

## DISCUSSION

In Kazakhstan CRC stay at third position among other cancers by estimated age-standardized incidence and mortality rates (14.3 and 7.6 per 100000 respectively) as for females, as for males (18 and 11.7 per 100 000 respectively) [3].

The genetic component of CRC is widely studied. According to publications, chromosome 10 is a carrier of polymorphisms associated with CRC [11, 12].

We successfully genotyped 12 SNPs on the 10th chromosome and found some evidence of association of 4 SNPs (rs10795668, rs1035209, rs11190164 and rs10506868) that were associated with CRC risk variation according to inheritance models.

A small number of studies describe these SNPs, nevertheless, the presented SNPs are demonstrated in research and play a role in changing the risk of CRC. These SNPs are likely involved in more sophisticated mechanisms of cancer development. A more detailed understanding of these patterns can help in a holistic view of the biological and genetic mechanisms associated with the initiation and progression of CRC.

Rs10795668 is leading to an aberrant expression of lncRNA by violating its regulatory region and modulate the binding of transcription factors to its promoter region. According to this mechanism regulation of the expression level of target genes occurs [13].

Rs10795668 is found by recent research to be related to family history CRC. A study by Gargallo et al. [14] showed that rs10795668 is associated with the susceptibility to colorectal adenomas and modified by the presence of a family history of CRC. The authors demonstrate that the low contribution of identified genetic variants associated with CRC does provide clinically relevant information by itself, but can increase the risk of CRC in a combination of risk-associated alleles in a polygenic model [14, 15].

More recently, some researchers used a genetic risk score, which adopts risk variants to predict CRC. Studies in Korea were based on seven SNPs, including rs10795668, to develop a genetic risk score for prediction and had significant associations with CRC and rectal cancer, but no clear association with colon cancer [16].

The Win et al. [17] observed that heterozygous and homozygous carriers of the G alleles for rs10795668 decreased CRC risk only among PMS2 mutation carriers. The authors also pointed out that these variants had the opposite direction observed for the general population [17]. A meta-analysis of genetic associations between SNPs and colorectal cancer risk published by Hong et al. [18], confirmed significant associations a risk variant at rs6983267 (OR: 1.388, 95% CI: 1.180–1.8633) which had the highest ORs in homogeneous model. Our study findings also indicate an increase in the risk of CRC in the presence of the G allele in the genotype of rs10795668.

Rs1035209 is located in an intergenic region of NKX2-3, SLC25A28. Lu et al. [19] demonstrated its role in regulating pathways of cells differentiation in CRC pointed that the imbalance between proliferation and differentiation in colorectal cells can cause cancer [20]. Whiffin and coauthors stated that their research has identified a novel CRC susceptibility SNP rs1035209 at 10q24.2 and it is associated with CRC risk in Europeans at genome-wide levels of significance [21]. A recent large-scale GWAS of the international group on 70,506 East Asians (22,775 cases and 47,731 controls) identified risk allele T of rs1035209 with OR from 1.04 to 1.12 (*p* = 3.6 × 10^−4^) [22]. An expressed risk of CRC in the presence of the effect T allele in SNP rs1035209 was determined in our study in Kazakhstan population.

Rs11190164 is located at a region containing multiple genes including SLC25A28, ENTPD7, COX15, CUTC, and ABCC2. A Genome-wide association study of Schumacher et al. on large groups (18,299 cases of colorectal cancer and 19,656 controls) from two Asian consortia showed a 9% increase in the odds of developing CRC per risk allele G in rs11190164 [8]. Our results also revealed the significant association with increased risk of CRC in the presence of the G allele in rs11190164. There were found associations of rs11190164 with rs1035209 and rs3740078 which were also significantly associated with CRC risk [21]. In addition, rs11190614 is highly linked with rs3740078 (r^2^ = 0.71, CEU population; *P* = 3.2E-05) which cause a synonymous mutation in the ENTPD7 gene leading to intestinal epithelial inflammation [23].

Rs10506868 is located in intron 6 of the VTI1A gene a fusion partner of a CRC susceptibility candidate gene, TCF7L2 [9]. It was found to be associated with the risk of colorectal cancer in East Asians and the association of this SNP with the risk of colorectal cancer needs to be further evaluated in other ethnic groups [9]. In our study, the presence of the effective allele T in rs10506868 leads to decrease the risk of colorectal cancer development in the population of the Republic of Kazakhstan. Zhang et al. study revealed strong evidence of the association with cancer risk for four variants (rs12241008, rs10506868, rs7086803 and rs11196067) in the VTI1A gene. The results demonstrate a weak correlation of rs10506868 with rs12241008 in Europeans (r^2^ = 0.201), moderate correlation in East Asians (r^2^ = 0.647), and no correlation in Africans (r^2^ = 0.014). The data of the presented article confirm the fact that the functional mechanisms of interaction of several SNPs associated with the risk of colorectal cancer may be opposite in different ethnic groups [24].

The study demonstrated the significant number of polymorphisms associated with colorectal cancer, located in the 10th chromosome in the Kazakhstan population. However, it is known, that the risk of CRC varies among individuals depending on the characteristics of diet, lifestyle and hereditary factors [25]. According to 2020 Global Nutrition Report [26] in Kazakhstan consumption of both red (59.9 g) and processed (19.3 g) meats overcome as regional (25.8 and 5.4 g respectively) as global values (31.4 and 12.2 g respectively). Unequal distribution of CRC cases over the country could be related with various dietary patterns in different parts of the country. According to Zhylkaidarova et al. [2] northern, eastern, and central regions with higher mortality rates have also often consumption of red meat. Dietary patterns in Western Kazakhstan include both meat and fish with medium mortality rates. Low rates were found in southern regions with traditional consumption of fruits and vegetables.

Thus, our findings demonstrate the prevalence of four polymorphisms in the 10th chromosome in the Kazakhstan population associated with sporadic CRC development. It is assumed that SNP interaction can vary in different ethnic groups and within environmental and personal risk factors may lead to alternative SNP patterns and its functional role in a share of pathology. The study of dietary and environmental influence on polymorphism allocation in the Kazakhstan population requires a more detailed analysis.

## MATERIALS AND METHODS

The study was performed with 282 CRC patients and 159 cancer-free controls living in the Karaganda Region, Kazakhstan. Each subject was interviewed by a doctor, and signed their written informed consents, afterwards participants donated 3–5 ml of venous blood. All participants underwent a general clinical examination at the outpatient stage, an express test of feces for occult blood, and fibrocolonoscopy as part of the national screening. The clinical diagnosis of CRC patients was established according to ICD 10. Criteria for inclusion: age 18–80 years with colorectal adenocarcinoma. Exclusion criteria: a hereditary form of CRC, somatic diseases in the stage of decompensation, the presence of genetic diseases. The research protocol was approved by the Committee on Bioethics of KSMU No. 305 dated May 19, 2017.

For genetic research, patients’ peripheral blood was taken into vacuum tubes with EDTA. Genomic DNA was extracted from whole blood using commercial kits GeneJET Genomic DNA Purification Kit (Thermo Scientific, Vilnius, Lithuania), in accordance with the manufacturer’s instructions. DNA concentration was measured using NanoPhotometer (Implen, Germany). The isolated DNA was stored at −20°C.

### SNP selection and genotyping

Selection and genotyping of SNPs were made using the Ensembl (https://www.ensembl.org/). We selected 12 candidate SNPs for genotyping panel, which previously reported being associated with CRC. Genotyping of SNPs located on chromosome 10 associated with CRC, were performed by QuantStudio 12K Flex PCR. PCR output was analyzed using an official ThermoFisher application (https://apps.thermofisher.com). Genotypes were determined in 2-dimensional space - a cluster of homozygous genotypes along the first allele was located near the X-axis, and homozygous genotypes along the second allele along Y. A cluster of heterozygous genotypes was formed between them.

### Statistical analyses

Statistical analysis was performed using online resource SNPstats (https://www.snpstats.net/) and R V 3.6.3 statistics. Based on the results of genotyping, for each polymorphism in two groups, such parameters as the percentage of major and minor alleles, the value of minor allele frequency (MAF), relative values for genotypes were calculated. Each SNP frequency was evaluated using Hardy-Weinberg equilibrium (HWE) with the exact criterion among controls. Comparison of genotypic distribution and allele frequencies between groups were performed using the χ^2^ test (Pearson’s chi-squared test). To estimate the association between the 10th chromosome SNPs and risk CRC, we measured odds ratios (ORs) and 95% confidence intervals (CIs). Differences between samples were considered statistically significant at *p* < 0.05. The genotype-phenotype association was determined using multiple inheritance models (dominant, recessive, and log-additive) to evaluate the associations between certain SNPs on the 10th chromosome and CRC. False discovery rate (FDR) adjustment was used for *p*-value.

## Abbreviations

CRC: colorectal cancer
SNP: single nucleotide polymorphism
OR: odds ratio
95% CI: 95% confidence interval
GWAS: genome-wide association study
HWE: Hardy–Weinberg equilibrium
MAF: minor allele frequency
EAS: East Asian population
SAS: South Asian population
AMR: American population
AFR: African population
FDR: false discovery rate
lncRNA: long non-coding RNA
NKX2-3: NK2 Homeobox 3
SLC25A28: Solute Carrier Family 25 Member 28
ENTPD7: Ectonucleoside Triphosphate Diphosphohydrolase 7
COX15: Cytochrome C Oxidase Assembly Homolog COX15
CUTC: Copper homeostasis protein cutC homolog
ABCC2: ATP Binding Cassette Subfamily C Member 2
VTI1A: Vesicle Transport Through Interaction With T-SNAREs 1A
TCF7L2: Transcription Factor 7 Like 2
ICD 10: International Classification of Diseases
PCR: polymerase chain reaction
